# Age-related changes of the tibial nerve cross-sectional area in the disabled elderly

**DOI:** 10.3389/fneur.2025.1721797

**Published:** 2026-01-26

**Authors:** Kholoud J. Sandougah, Mohamed A. Bedewi, Saeed M. Alamri, Nawaf A. Alzain, Abdulrahman A. Alharthi, Mohammed Ayesh Zayed, Mohamed Sherif El-Sharkawy, Saleh M. Alfawaz, Husain Alturkistani, Elsayed Abbas Beheri, Rasha Ali, Muneerah Al-Aqeel, Sitah F. Alanazi, Mohammad W. Marashdeh

**Affiliations:** 1 Department of Radiology, College of Medicine, Imam Mohammad Ibn Saud Islamic University (IMSIU), Riyadh, Saudi Arabia; 2 Department of Internal Medicine, College of Medicine, Prince Sattam bin Abdulaziz University, Al-Kharj, Saudi Arabia; 3 Department of Medical Rehabilitation, King Saud University Medical City, Riyadh, Saudi Arabia; 4 Department of Radiology & Medical Imaging, College of Medicine, King Saud University, Riyadh, Saudi Arabia; 5 Department of Family Medicine, Prince Sattam Bin Abdulaziz University Hospital, Al-Kharj, Saudi Arabia; 6 Arish university, College of sports science, Department of Training Sports and kinesiology, El-Arish, Saudi Arabia; 7 Department of Physics, College of Sciences, Imam Mohammad Ibn Saud Islamic University (IMSIU), Riyadh, Egypt

**Keywords:** cross sectional area, disabled, elderly, peripheral nerves, tibial nerve, ultrasound

## Abstract

**Objective:**

This study aims to assess changes in the cross-sectional area of the tibial nerve in the disabled elderly.

**Materials and methods:**

The study sample included 124 tibial nerves in 62 participants; 16 of which were disabled elderly patients (13 males, 3 females), with a mean age of 66.6, a mean height of 167.1 cm, a mean weight of 83.9 kg, and a mean BMI of 29.9. Twenty-three young controls (6 males, 17 females), a mean age of 48.4, a mean height of 154.9 cm, a mean weight of 79.8 kg, a mean BMI of 32.7, and 23 elderly non-disabled participants (13 males, 10 females), a mean age of 63.6, a mean height of 161.9 cm, a mean weight of 80.56 kg, and a mean BMI of 30.88.

**Results:**

The mean CSA of the TN in the elderly disabled group was 28.5 mm^2^. The mean CSA of the young control group’s TN was 20.45 mm^2^. The mean CSA of the TN in the elderly non-disabled group (both diabetic and non-diabetic) was 27.6 mm^2^. The mean CSA of the TN in the elderly diabetic non-disabled group was 29.9 mm^2^. The mean CSA of the TN in the elderly (non-diabetic) non-disabled group was 24.6 mm^2^.

**Conclusion:**

In conclusion, although our study suggests that nerve ultrasound could be a helpful tool for assessment of the tibial nerve in disabled elderly patients, disability status was not an independent predictor of tibial nerve CSA. Future studies with a larger sample size and a homogenous group are suggested.

## Introduction

Disability in the elderly population is a complex, multidimensional subject. The disabled elderly population face long-term physical, mental problems, and sensorimotor difficulties, which could cause partial or full restriction of social engagement (1–4). Many of these patients suffer from neuromuscular symptoms with weakness/atrophy of the skeletal muscles and/or changes in the peripheral nerve morphology. The presence of polyneuropathies, especially those related to chronic disease, can be disabling for the elderly (5). In the past, the diagnosis of chronic polyneuropathies was mainly based on clinical assessment and nerve conduction studies (6). Recently, musculoskeletal ultrasound has emerged as an efficient imaging tool in the diagnosis of different neuromuscular disorders (7, 8). The tibial nerve is one of the two branches of the sciatic nerve and was successfully used for the assessment of chronic peripheral nerve disease (9–12). Ultrasound is a cheap imaging tool with dynamic capability, without risk of ionizing radiation, with shorter acquisition time compared to MRI, and with the capability of bilateral examination (13). The cross-sectional area (CSA) is an important sonographic parameter for differentiation of normal and abnormal nerves (14). This study aims to assess the CSA changes of the tibial nerve in disabled elderly patients.

## Methods

### Participants

After institutional review board approval, participants for the study were recruited between February 2025 and May 2025, and written consent was obtained. A total of 16 elderly patients with different types of motor disability were recruited to a teaching university hospital. Inclusion criteria included elderly patients at least 60 years old with motor disabilities (DE). This group of four cases is due to motor disability due to cerebrovascular disease, four cases due to spinal acquired and congenital causes, five cases due to chronic peripheral vascular causes, two cases due to muscular degenerative and dystrophic diseases, and one case due to chronic peripheral nerve disease. Patients with temporary disability were excluded.

A second group included elderly patients at least 60 years old without motor disability, non-disabled elderly (NDE); these were subdivided into diabetic (type II) and non-diabetic. A nerve conduction study was performed on a quarter of the participants. The third group included healthy subjects below 60 years old. For each participant, data including age, sex, BMI, weight, and height were recorded.

### Technique

**Figure 1:**
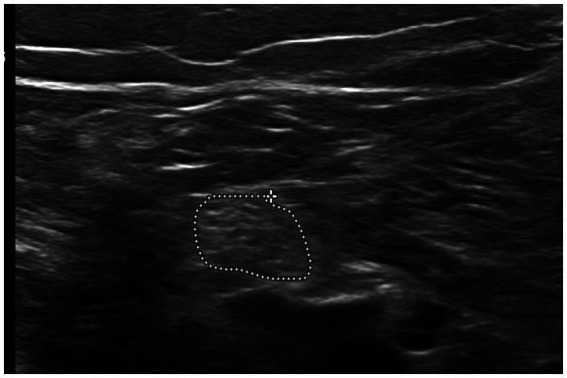
Short axis scan of the tibial nerve at the popliteal fossa, with measurement taken using the tracer method.

We used an L12–5 MHz linear transducer (Epic 7 version 1.5, Ultrasound system: Philips, Bothell, WA, USA). All participants were scanned by a radiologist (M.B.) with 13 years’ experience in neuromuscular ultrasonography. Each nerve was scanned three times, with the ultrasound probe removed from the skin in between. The tibial nerve was scanned in the popliteal fossa after division from the sciatic nerve and posterior to the popliteal artery and vein, and then the CSA was measured in mm^2^. The CSA of the TN was measured inside the hyperechoic epineurium using the tracer method; minimal pressure was exerted on the probe to optimize image quality (Figure 1).

### Statistical analysis

Statistical analysis was performed using Statistical Package for the Social Sciences (SPSS) version 27 software (SPSS Inc., Chicago, IL, USA). All data were presented as mean, standard deviation (SD), and range. The Shapiro–Wilk test was used to assess the normality of the data distribution. The correlations between the CSA of the scanned nerves, age, weight, height, and BMI were evaluated using Pearson’s correlation coefficient (*r*). A *p*-value of <0.05 was considered significant. The ANOVA test was used to compare CSA between different groups. An ANCOVA test was performed to control for confounding variables.

## Results

The study sample included 124 tibial nerves in 62 participants; 16 of them were disabled elderly patients (13 males, 3 females), mean age 66.6 ± 4.16, mean height 167.1 cm ± 9.2, mean weight 83.9 kg ± 13.1, mean BMI 29.9 ± 4.2; 23 young controls (6 males, 17 females), mean age 48.4 ± 5.8, mean height 154.9 cm ± 6.48, mean weight 79.8 kg ± 13.1, mean BMI 32.7 ± 4.2; and 23 elderly non-disabled participants (13 males, 10 females), mean age 63.6 ± 4.93, mean height 161.9 cm ± 9.11, mean weight 80.56 kg ± 15.88, and mean BMI 30.88 ± 5.3 (Table 1). The intra-observer reliability calculations resulted in an overall intra-class correlation coefficient of 0.88. The mean CSA of the TN in the elderly disabled group was 28.5 mm^2^ ± 9.9. The mean CSA of the young control group TN was 20.45 mm^2^ ± 7.9. The mean CSA of the TN in the elderly non-disabled group (both diabetic and non-diabetic) was 27.6 mm^2^ ± 8. The mean CSA of the TN in the elderly diabetic non-disabled group was 29.9 mm^2^ ± 9. The mean CSA of the TN in the elderly (non-diabetic) non-disabled group was 24.6 mm^2^ ± 5.1 (Table 2). The TN CSA in the non-disabled elderly group (NDE) showed moderate negative correlation with weight (*p* = 0.110) and BMI (*p* = 0.074). Otherwise, all three groups showed no significant statistical correlation with all other demographic factors (Table 3). One-way ANOVA and post-hoc Bonferroni test results are presented in Table 4.

**Table 1:** The demographic features of the study participants of the three groups in our study.

Variable	Control aged >60 (*n* = 23)		Control aged <60 (*n* = 23)		Elderly disabled (*n* = 16)		*p* value
	*N*	%	*N*	%	*N*	%	
Gender							
Male	13	56.52%	6	26.08%	13	81.25%	
Female	10	43.47%	17	73.91	3	18.75%	
Age (years)	(63.60 ± 4.93)		(48.43 ± 5.85)		(66.62 ± 4.16)		<0.001
Height (cm)	(161.91 ± 9.11)		(154.93 ± 6.48)		(167.12 ± 9.20)		<0.001
Weight (kg)	(80.56 ± 15.88)		(79.80 ± 14.64)		(83.91 ± 14.15)		0.306
Body mass index (kg/m^2^)	(30.88 ± 5.36)		(32.70 ± 5.73)		(29.98 ± 4.25)		0.211

SD, standard deviation.

**Table 2:** The mean tibial nerve CSA measured in square millimeter in different age groups.

Variable	Control aged >60 (*n* = 23)		DM (*n* = 10)		Non-DM (*n* = 13)		Control aged <60 (*n* = 23)		Elderly disabled (*n* = 16)		*p* value
	*N*	%	*N*	%	*N*	%	*N*	%	*N*	%	
	Mean± SD		Mean± SD		Mean± SD		Mean± SD		Mean± SD		
Cross sectional area	(27.66 ± 8.00)		(29.99 ± 9.05)		(24.64 ± 5.18)		(20.45 ± 7.97)		(28.55 ± 9.92)		<0.001

CSA, cross sectional area; DM, diabetes mellitus; SD, standard deviation.

**Table 3:** Correlation between TIB nerve, age, height, weight, and BMI in the disabled elderly group.

Correlation		TIB nerve	Age	Height	Weight	BMI
TIB nerve	Pearson correlation	1	0.025	−0.086	−0.414	−0.459
	Sig. (2-tailed)		0.927	0.750	0.110	0.074

**Table 4:** One-way ANOVA comparing TIB CSA between groups.

Group		Mean difference	Sig.	95% Confidence interval	
				Lower bound	Upper bound
G 1.00	2.00	7.19^*^	0.000	2.83	11.55
	3.00	−0.88	1.000	−5.73	3.96
G 2.00	1.00	−7.19^*^	0.000	−11.55	−2.83
	3.00	−8.07^*^	0.000	−12.97	−3.18
G 3.00	1.00	0.88	1.000	−3.96	5.73
	2.00	8.07^*^	0.000	3.18	12.97

* statistically significant differences *p* < 0.05.

## Discussion

In our study, the tibial nerve CSA in the disabled elderly group (28.5 mm^2^) was larger compared to the control young population group (20.45 mm^2^), and was also increased compared to other studies in the literature, such as Kerasnoudis et al. (8.43 mm^2^), Soek et al. (24.4 mm^2^), Fisse et al. (25.9 mm^2^) (15–17). The TN CSA in the elderly disabled group was also larger than the elderly non-disabled non-diabetic group (24.6 mm^2^); however, the elderly disabled group showed slightly lower values than the elderly diabetic non-disabled group (29.9 mm^2^). Analyzing our data with the ANOVA test and the ANCOVA model revealed that the difference in CSA between the disabled elderly group and the control young group was not statistically significant, and that none of the covariates (age, weight, height, BMI) could be significant predictors of tibial nerve CSA. This means that after controlling for confounding variables, the difference between these two was attenuated. Our results suggest an increase in CSA of the TN in disabled elderly patients in comparison to the control groups, except diabetic patients. The CSA of the TN in diabetic patients is reported in the literature to be increased in comparison to healthy controls. Diabetic patients with polyneuropathy are also reported to have higher CSA compared to both healthy controls and diabetic patients without polyneuropathy (18). The similarity in CSA (*p* < 0.05) between the elderly disabled group and the elderly diabetic non-disabled group could suggest a more influential role of aging and diabetes on CSA enlargement rather than disability itself as a sole factor. This study has several limitations. First, it is the small sample size. Second, a major limitation is the heterogeneity of the sample of the disabled elderly group, including a variety of patients with uneven distribution of nerve involvement. Third, examining the TN was challenging in some cases of disability due to the body habitus of some patients. In conclusion, although our study suggested that nerve ultrasound could be a helpful tool for assessment of the tibial nerve in disabled elderly patients, disability status was not an independent predictor of tibial nerve CSA. Future studies with a larger sample size and a homogenous group are suggested.

## Data Availability

The original contributions presented in the study are included in the article/supplementary material, further inquiries can be directed to the corresponding author.
